# Impact of Diabetes Perceptions on Medication Adherence in Japan

**DOI:** 10.3390/pharmacy7040144

**Published:** 2019-10-29

**Authors:** Koki Urata, Kana Hashimoto, Reiko Horiuchi, Kiichi Fukui, Kunizo Arai

**Affiliations:** 1 Faculty of Pharmacy, Institute of Medical, Pharmaceutical, and Health Sciences, Kanazawa University, Kanazawa 920-1192, Japan; 2 Gran Pharma Inc., 1-5-2 Hon-machi, Kanazawa 920-0853, Japan

**Keywords:** medication adherence, perception, community pharmacy services, diabetes mellitus, pharmaceutical care

## Abstract

**Background**: Patients’ perception of diabetes mellitus is one of the psychosocial factors influencing diabetic behavior. This patients’ perception of the disease is a mental image formed from the experience of patients with type 2 diabetes mellitus and reportedly reflects the aspect of recuperation. We investigated the relationship between changes in the patients’ perception of the disease and medication adherence, as influenced by the active involvement of community pharmacists. **Methods**: A prospective cohort study that used patient registry based in community pharmacies was conducted in patients with type 2 diabetes using oral antidiabetic agents at a pharmacy in Ishikawa Prefecture in Japan. Patients responded to the questionnaire at the time of enrollment and at the end of the one-year intervention period. The pharmacist confirmed the patient's medication status and treatment problems via telephone calls at least once every two weeks for one year. Main outcome measures: Type 2 diabetes patients’ perception of the disease related to medication adherence. **Results**: The study enrolled 113 patients. Among the seven diabetes image factors, “Living an orderly life” and “Feeling of fear” were significantly associated with medication adherence. “Feeling of neglect of health” was significantly associated at the subscale level. **Conclusion**: All the three factors related to medication adherence indicated self-care ability. To enhance the self-care ability of the patient, pharmacists should assist in self-care interventions for the patients.

## 1. Introduction

Diabetes is one of the most common chronic diseases, and the number of patients receiving diabetes-related drug treatment in Japan has been increasing over the years, reaching a record high of approximately 3.2 million according to the latest survey [1]. The clinical use of drugs with new action mechanisms has been widespread since 2010, and the glycemic control (HbA1c) situation has gradually improved. However, more than 40% of patients have yet to achieve the target HbA1c level of less than 7%, which may be associated with decreased adherence to therapies. 

Adherence is defined as the act of doing what is required by a rule, belief, etc. Compliance is the act or process of doing what you have been asked or ordered to do. This is the main difference between adherence and compliance. Therefore, adherence is not always good even when compliance is good. Poor adherence involves a combination of social and economic factors and patient internal factors that vary in each patient [2]. Several barriers and facilitators of adherence have been identified, including patient-related factors (belief, knowledge, cognitive function, and health literacy), physician-related factors (communication with patient), medication-related factors (adverse drug reaction, drug regimen complexity, and cost) and system-based factors (lack of medication review and lack of patient intervention) [3]. Various psychosocial factors are related to the self-management of taking medications in patients with chronic disease, and understanding the psychosocial factors involved in living with the disease is necessary for the continuation of support in taking medications [4,5]. The maintenance of good glycemic control is, in practice, very difficult for patients and health care professionals [6]. The major determinants of glycemic control are medical and behavioral factors, which include the insulin secretion, stage of disease, insulin resistance, the appropriateness of treatment, and diabetic self-care and self-management [7,8]. Another important determinant of glycemic control is psychological factors. Psychological factors influencing diabetic self-care and self-management include health beliefs self-efficacy [9], location of health controls [10], and emotions/images [11,12]. 

We focused on the patients’ perception of the disease as the psychological factor of the diabetic. The patients’ perception of the disease refers to the mental image in which the experience on having type 2 diabetes mellitus reflects the patients’ thoughts and the aspect of the medical treatment. By observing the diabetic patients’ perception of the disease, a new medical treatment approach could be found in the disease management. Kamatani et al. created a questionnaire for the purpose of understanding type 2 diabetes patients’ perception of their disease [13]. 

This study aimed to propose a strategy to solve the problem of medication adherence in patients with diabetes. The primary objective of this study was to examine the relationship between type 2 diabetes patients’ perception of the disease and the adherence to hypoglycemic medications. The secondary objective was to examine the relationships between pharmacist intervention and medication adherence. The results of the study could guide interventions directed at improving adherence.

## 2. Materials and Methods 

### 2.1. Study Design

This study is a prospective cohort study that used a patient registry based in community pharmacies [14]. A total of 62 pharmacists in 31 pharmacies, all of whom are members of the Aozora community pharmacies in Ishikawa Prefecture in Japan, voluntarily participated in this pilot study. 

### 2.2. Eligibility Criteria and Recruitment

Patients were included in this study if they were diagnosed with type 2 diabetes and used oral hypoglycemic medications (a list of the oral drug treatments available in Japan is shown in Appendix A). In Japan, the diagnosis of diabetes is made by the physician according to the Japanese Clinical Practice Guideline for Diabetes, etc. The diagnosis is not written on the prescription, but the pharmacist has access to the patient diagnosis by the patient interview. Inclusion criteria: Patients who had been prescribed with medications for 28 days or longer from hospitals, aged 40 years or older, and had a potential problem with their medical treatment were selected by community pharmacists. Exclusion criteria: Patients were excluded if they declined participation, or they could not be reached by telephone calls. 

Recruitment: Patients were recruited into the study at Aozora community pharmacies in Ishikawa Prefecture in Japan.

### 2.3. Study Procedure and Intervention

**Figure 1:**
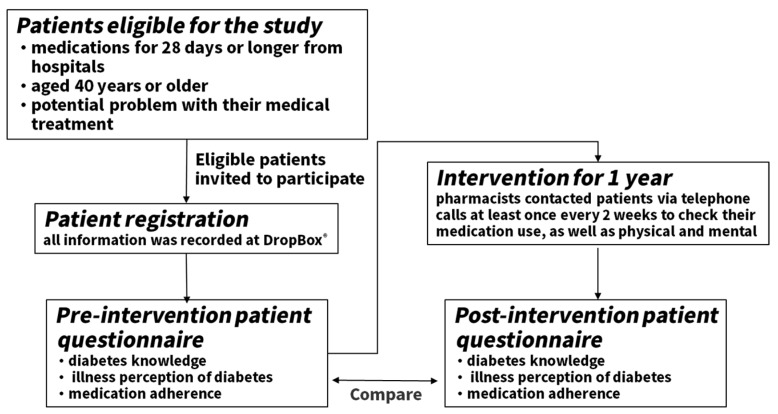
Overview of the practiced pharmacist intervention.

Pharmacists at participating pharmacies conducted research according to the following procedure (Figure 1):

Patient registration: To obtain cooperation from patients who conformed to the eligibility criteria, a pharmacist explained the research purpose and details of the research and obtained cooperation from the patients themselves for patient registration and intervention. 

Data collection at the initial assessments: Data, such as the patient registration ID, sex, age, clinical conditions for long-term medication use, date of the acquisition of the informed consent, and contact information if needed, were collected. Other data that were gathered were the list of all medications used by the patient (not only dispensed medication at the pharmacy but also those dispensed at different pharmacies), treatment history, other conditions that were not for long-term medication use, laboratory results (blood pressure, lipid, HbA1c, etc.), treatment conditions (one-pack doses, number of clinics used, etc.), living environments related to treatment (living alone, job, home care, etc.), potential problems reported by the patient, potential concerns identified by the pharmacist, and preferred methods for intervention contacts. The pharmacist accessed to the patient laboratory data by diabetes cooperation notebook, which was used for exchange of information between the clinics and hospital.

Pharmacist-led intervention: The data were collected by pharmacists from patient registration until the end of the monthly follow-up period, that is, one year for each patient. Contented changes in medication status, symptoms, laboratory results changes, and concerns about adverse reactions were recorded. Then, in between patients’ hospital visits, pharmacists contacted them via telephone calls at least once every two weeks to check their medication use, as well as physical and mental problems (as follow-up assessments). As described in detail at Appendix B**,** all information taken from the patients was recorded and shared among the pharmacists via an internet-based system (DropBox^®^). In the case of divided administration, confirmation at the time of arrival was made. To examine how the psychological aspect of patients with diabetes mellitus was changed by pharmacists who positively participated in the treatment, pharmacists conducted a questionnaire survey on medication adherence, type 2 diabetes patients’ perception of the disease, and knowledge on diabetes mellitus for all patients during registration and one year after [15]. When patients requested for withdrawal or had no further pharmacy visits, pharmacists asked the reasons at the last visit.

Data recording management: Data recording management was performed in the following steps by using DropBox^®^. Pharmacists recorded patient information by using Excel sheets at initial and intervention assessments. The data was anonymized before they were uploaded to DropBox^®^ and protected by a password.

Patient registration period: Patient registration lasted for one year (October 2016 to September 2017), and the intervention period was one year after.

Sample size: The sample size in the study was based on the justification according to Kamatani et al. [13]. An accuracy of 0.05 can be interpreted as the expected accuracy of the classifier to be within 5% of the "best" possible accuracy achieved with a binary classifier. Moreover, with 10-fold cross validation, we expect that power will be high (over 80%) with a two-sided type I error of 5% with the expected 50 patient target accrual.

Data collection of diabetes knowledge, illness perception of diabetes and medication adherence: We used the revised Michigan Diabetes Knowledge Scale (MDKT) [16] to measure the patients’ knowledge of diabetes treatment. The test has two components: a 14-item general test and a 9-item insulin use subscale. The report showed the reliability and validity of the Diabetes Knowledge Test. The questionnaire for type 2 diabetes patients’ perception of the disease, developed by Kamatani et al. [13], was used to collect data on the participants’ perceptions of their diabetes. The questionnaire consisted of 28 items and seven factors. Subjects were classified into three clusters depending on the seven factors of their perceptions of diabetes. And medication adherence was assessed using the Ueno method [17]. Ueno et al. developed the 12-item medication adherence scale for patients with chronic disease in Japan. The scale was categorized into four factors: “medication compliance”, “collaboration with healthcare providers”, “willingness to access and use information about medication”, and “acceptance to take medication and how taking medication fits patient’s lifestyle”. The items that can help understand the knowledge on treatment with the help of healthcare providers and the knowledge on treatment for its long-term continuation in the patient’s daily life and lifestyle management conditions, apart from “compliance” as a medication adherence scale in chronic diseases [13]. The reliability and validity of this questionnaire were reported (Appendix C).

### 2.4. Outcome Measures

The primary outcome measure was the changes in diabetes knowledge, illness perception of diabetes, and medication adherence during the year of intervention. The secondary outcomes measured during the year of intervention were (1) the number of telephone calls by pharmacists and (2) change in HbA1c.

### 2.5. Statistical Procedures and Analyses

All analyses used nonparametric methods. Quantitative variables were expressed as interquartile range (IQR) values. Moreover, Mann–Whitney's U test was used for independent comparisons between the two groups. SPSS version 25 was used for statistical analysis, with a significance level of 5% for both sides.

### 2.6. Ethics Approval

The study was conducted after being approved by the Ethics Committee at Kanazawa University in Japan (57-1, 17 September 2016). The participants were explained the purpose of the study, assured confidentiality of the collected data, and asked to sign a written consent to participate in the study. 

## 3. Results

**Figure 2:**
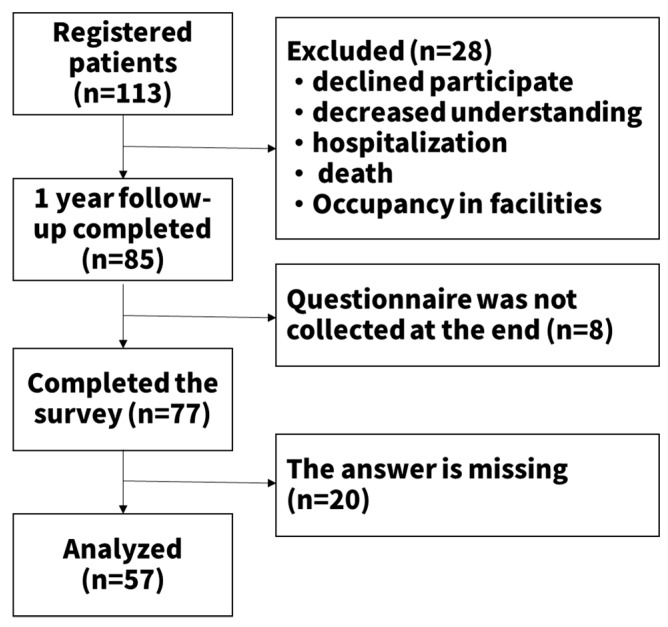
Participant recruitment flow chart.

The study enrolled 113 patients with type 2 diabetes. Among them, 85 completed the one-year intervention. Furthermore, 28 persons withdrew for reasons such as no visit of the pharmacist, impaired comprehension, changes in hospital, moving into nursing a home, and death. Of the 85 patients, 77 completed and returned the questionnaires. Of these 77, 20 were excluded from the analysis because the questionnaire was not completed. Finally, the data of 57 persons were analyzed (Figure 2). The sensitive patient population was characterized by a median age of 70 years (IQR, 63.5–77.0), a duration of illness of 10 years (IQR, 5–23.5), median HbA1c of 7.0 (IQR, 6.5–7.6), and the medication number was 5 (IQR, 3–7) (Table 1).

There was no statistically significant difference between pre- and post-intervention in medication adherence, type 2 diabetes patients’ perception of the disease, knowledge of diabetes mellitus (MDKT score), and HbA1c (Table 2). We conducted post hoc analysis stratifying for three levels of type 2 diabetes patients’ perception of the disease, namely increased, unchanged, and decreased, to examine the relation in medication adherence with type 2 diabetes patients’ perception of the disease. Table 3 presents the changes in medication adherence in the group with type 2 diabetes patients’ perception of the disease. Medication adherence was related with patients’ perception of Factor 2, “Living an orderly life”, and Factor 7, “Feeling of fear.” Furthermore, significant differences were observed in changes in Factor 6, “Feeling of neglect of health”, at the subscale level of adherence (Table 4).

We examined the pharmacist interventions made regarding medication use in patients with diabetes in the clinical trial setting. The frequency of interventions by pharmacist did not significantly correlate with any of the following: medication adherence, type 2 diabetes patients’ perception of the disease, knowledge of diabetes mellitus (MDKT score), and HbA1c (data not shown).

## 4. Discussion

We conducted a cohort study of 113 patients with type 2 diabetes mellitus. We found medication adherence was related with patients’ perception of Factor 2, “Living an orderly life”, and Factor 7, “Feeling of fear.” Furthermore, significant differences were observed in changes in Factor 6. However, there was no statistically significant difference between pre- and post-intervention in medication adherence, type 2 diabetes patients’ perception of the disease, knowledge of diabetes mellitus (MDKT score), and HbA1c by pharmacists' telephone consulting.

The reviewed systematic reviews indicating that factors associated with medication adherence in patients with diabetes are multifactorial with remarkably consistent findings across the reviews [18]. Barriers to or factors associated with medication adherence derived from the included reviews were categorized into the following: patient-related factors (demographic characteristics (age, sex, ethnicity, financial status and level of income, marital status, and level of education), physiological status (comorbidities, depression, smoking, and forgetfulness), health literacy (lack of understanding about the disease and treatment and difficulty reading the prescription), emotions (blame, guilt, shock and helplessness, frustration, negative attitude, stress, and anxiety), fears (injection, blood phobia, and fear of pain), perceptions of (need of medicine, barriers to follow medication, benefit from treatment, misconception about medications, and self-efficacy), adaptation to change (traveling overseas, alterations in daily schedule, change or lack of routine in managing treatment, and diet adjustments)); medication-related factors (frequency of dose or injection, length of therapy, number of medications and polypharmacy, timing of dosing, changing of treatment, fluctuating response to medications, side effects, complexity of regimen, drug class/type, method of drug administration, traditional medicine, and phytotherapy); disease-related factors (diabetes duration, disease complexity, lower HbA1c , and complications); provider-related factors (support from healthcare providers, patient not included in decision-making process, duration of counseling and lack of time, relationship with care provider, assumptions by providers about the patients’ knowledge, providing ambiguous or incomplete information, provider’s lack of experience, language and communication barrier); societal-related factors (support from family, lack of support, cultural barriers, and stigma); healthcare system–related factors (insurance coverage, lack of guide lines about optimal treatment, cost of medicine, co-payment amount, convenience of obtaining medications, and continuity of care); and HbA1c.

Medication adherence is affected by patients’ psychosocial factors that may be implicated in diabetes mellitus self-management [9,10,11,12]. Modifiable targets of psychological intervention are presented across the following three overarching domains: (1) knowledge, beliefs, and related cognitive constructs; (2) emotional distress and wellbeing; and (3) behavioral skills and coping. Knowledge, beliefs, and related cognitive constructs regarding their illness and treatment are predictive of long-term medication adherence [19,20,21,22,23,24,25,26,27,28,29].

Kamatani et al. reported that the patients’ perception of the disease as the psychological factor of the diabetic was consistent with the seven factors of their perceptions. The seven factors of their perceptions of diabetes were correlated to the diabetes-related burden and the self-care agency. Factor 1, “Feeling of misery”, Factor 3, “Feeling of restriction”, Factor 4, “Feeling of misery”, and Factor 5, “Feeling of getting into trouble” of perceptions of diabetes were related to the diabetes-related burden of the patient. In addition, Factor 2, “Living an orderly life”, Factor 6, “Feeling of neglect of health”, and Factor 7, “Feeling of fear” were associated with the medical treatment life on self-care, especially health care [13].

We previously examined the relationship between type 2 diabetes patients’ perception of the disease and the adherence to hypoglycemic medications. In a cross-sectional analysis using a questionnaire survey, Factor 2, “Living an orderly life”, showed a significant positive correlation with adherence [15]. In the current study, we examined the relationship between the patients’ perception of the disease and medication adherence by the amount of change in each factor at one-year intervention. The adherence was significantly improved in patients with increased Factor 2, “Living an orderly life” and Factor 7, “Feeling of fear.” Factor 6, the “Feeling of neglect of health” score was significantly correlated with the subscale of medication adherence. All of the patients’ perception of the disease associated with these adherences are factors related with self-care ability. Therefore, pharmacists should take an active role in providing diabetes self-care education in conjunction with euglycemic medicines, in order to improve patient outcomes. The feasibility of medication adherence relies on support through patient counseling, peer education, and health interventions, all of which needed in Japanese primary care settings.

Understanding the changes in physical conditions in a timely manner was difficult, with confirmation only at the time of administration for long-term prescription patients who had long visit intervals. However, to fulfill the primary care functions of pharmacists, they need to confirm whether pharmacotherapy is safely continued even at times other than the next visit and to provide information to the physician. Various studies have suggested that telephone monitoring as an intervention method of the pharmacist could improve patient adherence as well as their health and economic outcomes [4,30,31,32,33,34]. In this study, through telephone monitoring, health problems after the change of prescription and aggravation of side effects were detected, but it was not related to the improvement of medication adherence or improvement of glycemic control. The lack of change in patient adherence toward pharmacist intervention may be because of the high initial adherence of patients recruited in this study (full marks on the lower-scale Factor 4: medication compliance) or because the telephone monitoring as the pharmacists’ intervention is not beneficial for the improvement in the patients’ self-care ability.

The common-sense model of self-regulation is a health-specific model that examines the cognitive and emotional activities that occur throughout the chronic illness experience. The model may be used to help clinicians develop appropriate interventions by gaining an understanding of human efforts to protect health and reduce the threat caused by chronic illness [5]. Low levels of diabetes self-care execution are associated with patients’ deficiency in self-regulatory resource, and self-care as a series of goal-directed behaviors consumes patients’ self-regulatory resources before these behaviors becomes a habit [35].

## 5. Limitations

The data derived for the study was limited to one pharmacy group in Kanazawa and restricted to the outpatient clinic for diabetes. Self-reported assessment of medication adherence may be over-estimated by patients. Face-to-face interviews and use of self-reports might generate socially desirable answers. Many variables were unavailable for study inclusion, such as the severity of type 2 diabetes, information on dietary habits, daily exercise assessment, and laboratory data. Assessment of these factors might provide a clearer picture about diabetes knowledge, medication adherence, and glycemic control. In addition, it has been most frequently observed that adolescents who are nonadherent are less likely to take part in research. Therefore, we are uncertain that those who consented to participate were those with better adherence or vice versa. We also could not evaluate the extent to which the nature of conversations or counseling recommendations differed across the arms, because we did not have access to deidentified versions of the pharmacist consultations.

## 6. Conclusions

We found medication adherence was related with patients’ perception of Factor 2, “Living an orderly life”, and Factor 7, “Feeling of fear.” Furthermore, significant differences were observed in changes in Factor 6. All of the patients’ perception of the disease associated with these adherences are factors related with the self-care ability. Therefore, pharmacists should provide interventions that enhance patient’s self-care capacity and build a more therapeutically effective patient–pharmacist relationship.

## Figures and Tables

**Table 1:** Characteristics of patients with type 2 diabetes at study entry.

Characteristics of Patients		Total (n = 57)	
		n	%
Gender (n)	male	35	61.4
	female	22	38.6
Age (year)	median (IQR, 25th–75th percentile)	70 (63.5–77.0)	
BMI	median (IQR, 25th–75th percentile)	23.3 (21.5–25.1)	
Diabetes duration (year)	median (IQR, 25th–75th percentile)	10 (5.0–23.5)	
HbA1c (%)	median (IQR, 25th–75th percentile)	7.0 (6.5–7.6)	
Diabetes history of relatives	yes	31	54.4
	no	26	45.6
Complications (n)	retinopathy	5	
	nephropathy	2	
	neuropathy	4	
	cardiovascular	5	
	none	41	
Therapy (n)	exercise therapy	13	
	diet therapy	20	
	insulin therapy	10	
Number of medications (n)	median (25–75 percentile)	5 (3–7)	

**Table 2:** Changes in medication adherence, type 2 diabetes patients’ perception of the disease, the Michigan Diabetes Knowledge Scale (MDKT) score and HbA1c during a one-year intervention.

	Pre	Post	*p* ^a)^
Medication Adherence Total score (out of 75 points)	53.0 (47.5–60.0)	57.0 (51.0–62.0)	0.138
Type 2 diabetes patients’ perception of the disease score			
Factor 1. Feeling of inferiority	3.00 (1.00–5.00)	2.80 (1.20–4.60)	0.629
Factor 2. living an orderly life	6.67 (5.50–8.33)	6.67 (5.00–81.17)	0.562
Factor 3. Feeling of restriction	5.80 (4.80–6.60)	6.00 (4.40–7.10)	0.667
Factor 4. Feeling of misery	4.67 (3.00–6.17)	4,67 (3.33–6.50)	0.409
Factor 5. Feeling of getting into trouble	4.20 (2.70–5.00)	4.40 (3.10–5.60)	0.346
Factor 6. Feeling of neglect of health	6.67 (4.50–7.67)	6.00 (4.50–7.67)	0.362
Factor 7. Feeling of fear	6.33 (3,67–7.50)	6.00 (5.00–7.00)	0.708
MDKT score Total score (out of 14 points)	7.0 (5.6–9.4)	7.8 (5.5–9.4)	0.818
HbA1c (%)	7.0 (6.5–7.6)	7.4 (6.5–7.8)	0.118

a) Mann–Whitney U test.

**Table 3:** Relationship between medication adherence and type 2 diabetes patients’ perception of the disease.

Type 2 Diabetes Patients’ Perception of the Disease Score	Increased Group	Unchanged Group	Decreased Group	*p* ^a)^
	Change of Medication Adherence Score ^b)^			
Factor 1. Feeling of inferiority	0 (−5.5–5.5)	1.0 (−3.3–8.0)	2.0 (−3.0–9.0)	0.308
Factor 2. living an orderly life	3.5 (0.0–8.8)	−2.0 (−2.0–5.0)	−2.0 (−7.5–4.0)	0.028 *
Factor 3. Feeling of restriction	1.0 (−3.0–12.0)	1.0	2.0 (−5.0–8.0)	0.678
Factor 4. Feeling of misery	4.0 (−2.0–9.0)	1.0 (−5.0–1.5)	−1.0 (−5.5–6.5)	0.173
Factor 5. Feeling of getting into trouble	2,5 (−2.0–8.0)	−2.0	−0.5 (−6.5–8.0)	0.220
Factor 6. Feeling of neglect of health	4.0 (−1.0–12.5)	1.0 (−1.0–5.0)	−1.5 (−6.3–6.5)	0.062
Factor 7. Feeling of fear	5.0 (−0.5–10.5)	−3.0	−0.5 (−6.8–4.0)	0.015 *

a) Mann–Whitney U test. b) Values are presented as median (IQR, 25th-75th percentile). * significant at 0.05 level

**Table 4:** Association between a subscale of medication adherence and Factor 6, “Feeling of neglect of health”.

	Change of Medication Adherence Score ^b)^			
Medication Adherence Subscale	Increased group in Factor 6, “Feeling of neglect of health” (n = 22)	Unchanged group in Factor 6, “Feeling of neglect of health” (n = 5)	Decreased group in Factor 6, “Feeling of neglect of health” (n = 30)	*p* ^a)^
Factor 1: Relationship between patient and medical staff	1.5 (0–3.5)	0.0 (−0.5–0.5)	0.0 (−2.3–2.0)	0.308
Factor 2: Medication information gathering	3.0 (−1.0–6.3)	1.0 (0.0–1.5)	0.0 (−3.3–3.3)	0.028 *
Factor 3: Behavioral and motivational about medication	0.5 (−1.0–2.0)	1.0 (−1.0–2.0)	−0.5 (−2.0–0.0)	0.678
Factor 4: Medication compliance	0.0 (−1.3–1.0)	1.0 (−1.0–2.0)	0.0 (0.0–2.0)	0.173
Total score	4.0 (−1.0–12.5)	1.0 (−1.0–5.0)	−1.5 (−6.3–6.5)	0.220

a) Mann–Whitney U test. b) Values are presented as median (IQR, 25th–75th percentile). * significant at 0.05 level

## Appendix A

**List of Oral Drug Treatment Used in Japan**

	**Generic Name**
Biguanide	Metformin
	Buformin
Thiazolidinediones	Pioglitazone
Sulfonylureas (SU) drugs and related drugs	Glibenclamide
	Gliclazide
	Glimepiride
Rapid-acting insulin secretagogues	Nateglinide
	Mitiglinide
	Repaglinide
DDP-4 inhibitors	Sitagliptin
	Vildagliptin
	Alogliptin
	Linagliptin
	Teneligliptin
	Anagliptin
	Saxagliptin
	Trelagliptin
	Omarigliptin
Alpha-glucosidase inhibitors	Acarbose
	Voglibose
	Miglitol
SGLT2 inhibitors	Ipragliflozin
	Dapagliflozin
	Luseogliflozin
	Canagliflozin
	Empagliflozin
Combination drug	Pioglitazone/Metformin
	Pioglitazone/Glimepiride
	Pioglitazone/Alogliptin
	Mitiglinide/Voglibose
	Vildagliptin/Metformin

## Appendix B

**Data Collection at Intervention Assessments**

1. Date of contacts
2. Method for monitoring (telephone or visit)
3. Changes in medications
4. Patient reported adherence or number of medications not used
5. Reason for not taking medications as instructed
6. Conditional changes (if any)
7. Laboratory results (reported by patients)
8. Pharmacist advice given to patients
9. Next appointment dates

When patients request a withdrawal, or have no further pharmacy visits, pharmacists should ask the reasons at the last visit or try to contact patients or family members as much as possible.

## Appendix C

**Medication Adherence Scale 14-item Version**

Instructions

This is a survey about your use of the medication that is currently prescribed for you. (Please answer all items 1–14) Unless otherwise specified, please base your answer on your experiences over the last six months or so. If you suffer from multiple conditions, your response should reflect your overall usage of medication.

*Note: In this survey, “medication” includes medicine administered orally, injections (insulin, for example), ointments, medicated patches, and inhalants.

To what extent do these apply? (circle the one that most applies) (never ~ always)

	**Never**	**Rarely**	**Sometimes**	**Often**	**Always**
Items (1)–(14)	1	2	3	4	5

Questions 1–14

I. Relationship with my healthcare provider with regard to medication

(1) I can freely ask my healthcare provider about medication without constraint.

(2) I can share my thoughts and goals about medication with my healthcare provider.

(3) I can share my past course of treatment with medication with my healthcare provider.

II. Collecting and using medication-related information

(4) I have asked about anything I do not understand about the medication I am using.

(5) I report side effects, allergic reactions, or unusual symptoms caused by medication.

(6) I know about the medication I am using and the need to take it.

(7) I am taking measures to continue medication (taking measures on a daily basis, etc.).

(8) I am searching for and using the information necessary for my medication.

III. Ideas and attitude towards medication

(9) I am convinced that medication is necessary.

(10) Taking medication is part of my everyday life, just like eating or brushing my teeth.

(11) I am not resistant to receiving help from family and people around me, such as reminding me to take my medication.

IV. Current status of medication use (please respond regarding use during this 3-week period)

(12) Over the past three weeks, I have been taking medication at the prescribed daily dosage.

(13) Over the past three weeks, I have been taking medication at the specified time/s.

(14) I will not stop taking medication based on my own judgment.

**Patients’ Perceptions of Diabetes Questionnaire**

The following are 28 statements about patients’ perceptions of diabetes. Please read each statement and rank your response on the 11-point scale directly below each statement, with “0” indicating that you strongly disagree with the statement and “10” indicating that you strongly agree with the statement.

(1) The complications of diabetes seem horrible.
(2) Fat people get diabetes.
(3) Sweets and greasy foods may cause diabetes.
(4) A specific lifestyle may cause someone to get diabetes.
(5) The body will gradually lose mobility because of the complications of diabetes.
(6) Diabetes causes me to feel bad.
(7) Diabetes does not cause harm.
(8) Diabetes is a terrible disease.
(9) Diabetes is not an illness for which one dies alone.
(10) All I can do is not make it worse.
(11) I cannot eat anything the same way as another person.
(12) Diabetes causes sorrow.
(13) Diabetes is determined by our genetics.
(14) I am not motivated to treat my diabetes properly.
(15) Insulin-requiring states for treating diabetes are severe.
(16) I feel that I will waste all of my life when I think about having/living with diabetes.
(17) I feel embarrassed about my diabetes.
(18) Diabetes may cause serious trouble for me.
(19) I feel incompetent as a man with diabetes.
(20) Another person has no reason to complain about my diabetes.
(21) I am labeled a failure.
(22) I feel alone with diabetes.
(23) I am grateful for my situation having/living with diabetes.
(24) I feel that having/living with diabetes is my destiny.
(25) I cannot understand diabetes.
(26) Diabetes is a bad friend.
(27) Daily routines are regulated by having/living with diabetes.
(28) I feel quite safe when I manage my diabetes well.
(29) I am constantly concerned about food and eat

**Revised Michigan Diabetes Knowledge Scale (DKT)**

Here are 20 statements about diabetes, some are true statements and some are false. Please read each statement and then indicate whether you think it is true or false by putting a circle round either TRUE or FALSE. If you do not know the answer please put a circle around DON’T KNOW.

1. The diabetes diet is a healthy diet for most people

TRUE / FALSE / DON’T KNOW

2. Glycosylated haemoglobin (HbA1c) is a test that measures your average blood glucose level in the past week.

TRUE / FALSE / DON’T KNOW

3. A pound of chicken has more carbohydrate in it than a pound of potatoes.

TRUE / FALSE / DON’T KNOW

4. Orange juice has more fat in it than low fat milk.

TRUE / FALSE / DON’T KNOW

5. Urine testing and blood testing are both equally as good for testing the level of blood glucose.

TRUE / FALSE / DON’T KNOW

6. Unsweetened fruit juice raises blood glucose levels.

TRUE / FALSE / DON’T KNOW

7. A can of diet soft drink can be used for treating low blood glucose levels.

TRUE / FALSE / DON’T KNOW

8. Using olive oil in cooking can help lower the cholesterol in your blood.

TRUE / FALSE / DON’T KNOW

9. Exercising regularly can help reduce high blood pressure.

TRUE / FALSE / DON’T KNOW

10. For a person in good control, exercising has no effect on blood sugar levels.

TRUE / FALSE / DON’T KNOW

11. Infection is likely to cause an increase in blood sugar levels.

TRUE / FALSE / DON’T KNOW

12. Wearing shoes a size bigger than usual helps prevent foot ulcers.

TRUE / FALSE / DON’T KNOW

13. Eating foods lower in fat decreases your risk for heart disease.

TRUE / FALSE / DON’T KNOW

14. Numbness and tingling may be symptoms of nerve disease.

TRUE / FALSE / DON’T KNOW

15. Lung problems are usually associated with having diabetes.

TRUE / FALSE / DON’T KNOW

16. When you are sick with the flu you should test for glucose more often.

TRUE / FALSE / DON’T KNOW

17. Having regular check-ups with your doctor can help spot the early signs of diabetes complications.

TRUE / FALSE / DON’T KNOW

18. Attending your diabetes appointments will stop you getting diabetes complications.

TRUE / FALSE / DON’T KNOW
